# Bioactive Phytoconstituents and Their Therapeutic Potentials in the Treatment of Haematological Cancers: A Review

**DOI:** 10.3390/life13071422

**Published:** 2023-06-21

**Authors:** Emeka J. Iweala, Adurosakin E. Oluwapelumi, Omoremime E. Dania, Eziuche Amadike Ugbogu

**Affiliations:** 1 Department of Biochemistry, College of Science and Technology, Covenant University, Ota PMB 1023, Ogun State, Nigeria; 2 Covenant Applied Informatics and Communication African Centre of Excellence (CApIC-ACE), Covenant University, Ota PMB 1023, Ogun State, Nigeria; 3 Department of Microbiology, Ladoke Akintola University of Technology, Ogbomoso PMB 4000, Oyo State, Nigeria; 4 Department of Biochemistry, Abia State University, Uturu PMB 2000, Abia State, Nigeria

**Keywords:** bioactive compounds, haematological cancers, anti-cancer mechanisms, anti-inflammatory, anti-oxidative, chemotherapy

## Abstract

Haematological (blood) cancers are the cancers of the blood and lymphoid forming tissues which represents approximately 10% of all cancers. It has been reported that approximately 60% of all blood cancers are incurable. Despite substantial improvement in access to detection/diagnosis, chemotherapy and bone marrow transplantation, there is still high recurrence and unpredictable but clearly defined relapses indicating that effective therapies are still lacking. Over the past two decades, medicinal plants and their biologically active compounds are being used as potential remedies and alternative therapies for the treatment of cancer. This is due to their anti-oxidant, anti-inflammatory, anti-mutagenic, anti-angiogenic, anti-cancer activities and negligible side effects. These bioactive compounds have the capacity to reduce proliferation of haematological cancers via various mechanisms such as promoting apoptosis, transcription regulation, inhibition of signalling pathways, downregulating receptors and blocking cell cycle. This review study highlights the mechanistic and beneficial effects of nine bioactive compounds (quercetin, ursolic acid, fisetin, resveratrol, epigallocatechin gallate, curcumin, gambogic acid, butein and celastrol) as potential remedies for chemoprevention of haematological cancers. The study provides useful insights on the effectiveness of the use of bioactive compounds from plants for chemoprevention of haematological cancers.

## 1. Introduction

**Figure 1:**
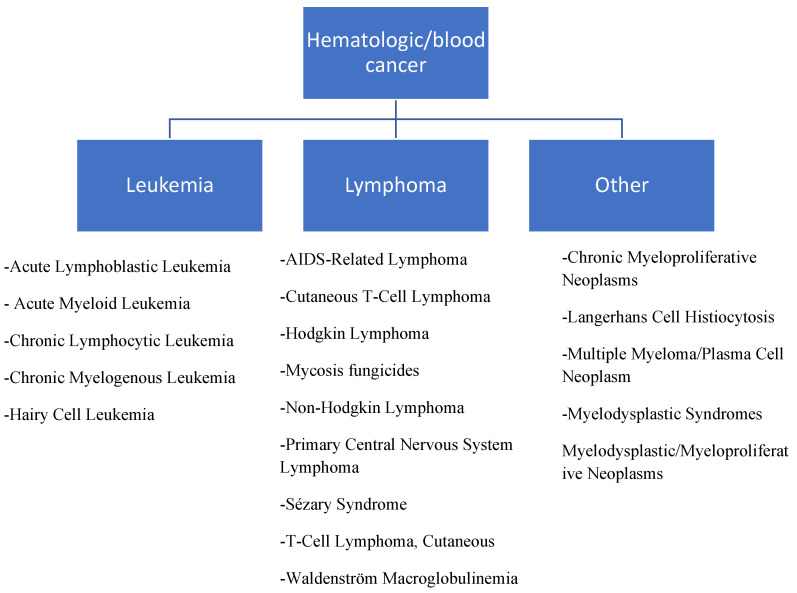
Types of haematological cancer according to the National Cancer Institute (NCI).

Worldwide, cancer is the second most leading cause of death and a public health challenge. It is a devastating, life-threatening and potentially traumatic disease; its fatality rate depends on its various types. Haematological malignancies or haematological cancers constitute a group of diseases characterised by an uncontrollable or abnormal growth of blood cells and their accumulation in the bone marrow [1,2]. According to the US National Cancer Institute, haematological cancers are divided into 2 main classes: leukaemia and lymphoma (Figure 1). Leukaemia is a board term for cancers of the blood cells; they are classified based on the blood cell type and how fast they develop. Leukaemia has a 5-year survival rate of 65.7%. Lymphomas on the other hand, are cancer of the lymph system with a 73.8% relative survival rate. Treatment options for haematological cancers include chemotherapy, radiation therapy, targeted therapy, immunotherapy, surgery and chemotherapy with stem cell transplant [3]. While the pathophysiology of various cancers deals with the development of tumours in the organs, haematological cancers are found in the bone marrow or lymphatic system. This has made it difficult to effectively administer therapeutic doses of anti-cancer agents to these sites.

According to the World Health Organization, the classification of haematological malignancies into different categories is based on the origin of the uncontrollably differentiating cell, the stage of disease development and clinical features of the condition [4,5]. In the last few years, intensive chemotherapy and bone marrow transplantation have been employed to treat patients suffering from different forms of cancers, including haematological malignancies. However, in chemotherapy, there has been less than 50% survival rate [6] with numerous side effects such as organ failure [7], immunosuppression [8] and most commonly, drug resistance [9]. Additionally, bone marrow transplantation has been associated with high risk of complications and is usually performed only in adults [10,11]. Alternatively, targeting of different cancer hallmarks such as differentiation, proliferation and apoptosis which contribute to malignancy has also been explored [12]. However, due to the complexity of the disease condition and the insufficient knowledge as to its underlying mechanism, these approaches have not been effective. Therefore, this has necessitated the development of newer therapeutic options which would improve already existing ones and result in better patient outcomes.

According to Gopal et al. [13], there will be an increase of 75% incidence and mortality from haematological cancer in sub-Saharan Africa by 2030. This could be attributed to the fact that 36% of cancer in Africa is as a result of infections such as HIV, EBV, malaria and Kaposi sarcoma-associated herpes virus (KSHV). These infections have been associated with different types of haematological cancer [14,15,16,17]. Majority of the health centres with infrastructure and manpower to diagnose, manage and/or treat cancer are mainly located in major cities and out of reach for majority of the population. This, and considering the high cost of treatment, makes most patients subscribe to traditional treatments.

Bioactive compounds which are naturally obtained from plants have been recognised for their therapeutic potential in the treatment of different types of cancers [18,19,20,21,22]. These naturally sourced products, which are sometimes referred to as herbs or traditional medicines, have been recognised for their roles as chemotherapeutic adjuvants [9,23,24]. They perform this function by acting in synergy with the drug to improve its efficacy, thereby creating better outcomes than when the drugs are used in isolation. In this regard, these bioactive phytoconstituents are used in combination therapies with chemotherapeutic drugs to improve the sensitivity of the cancer cells to the drugs either by optimizing drug functions or reducing dosage required which minimises toxicity [9,25]. Additionally, these bioactive compounds have also been examined in isolation or in combination with other bioactive compounds to address haematological malignancies [26].

So far, a good deal of evidence exists as to the beneficial roles of these bioactive phytoconstituents in the treatment of haematological malignancies. Therefore, there is continuous research on the development of new cancer therapeutics from these traditional compounds or phytochemicals. The selected phytochemicals reviewed in this study are polyphenols. The literature is rich with reports of the potential of polyphenols to protect against chronic diseases. Polyphenols are good anti-oxidants with pro-apoptotic and anti-cancer properties. They can modulate cell cycle signalling pathways and remove anti-cancer agents [27,28,29].

The efficacy of some common compounds such as curcumin, resveratrol, quercetin in the treatment of leukaemia, human multiple myeloma has been explored [30,31,32,33,34]. Therefore, this article presents a review of nine (9) bioactive phytoconstituents whose potential have been examined in the treatment of haematological malignancies. The review encompasses the chemical components of these bioactive compounds, their mechanism of action in different treatments under different conditions and the outcome of treatments when compared to existing solutions.

**Figure 2:**
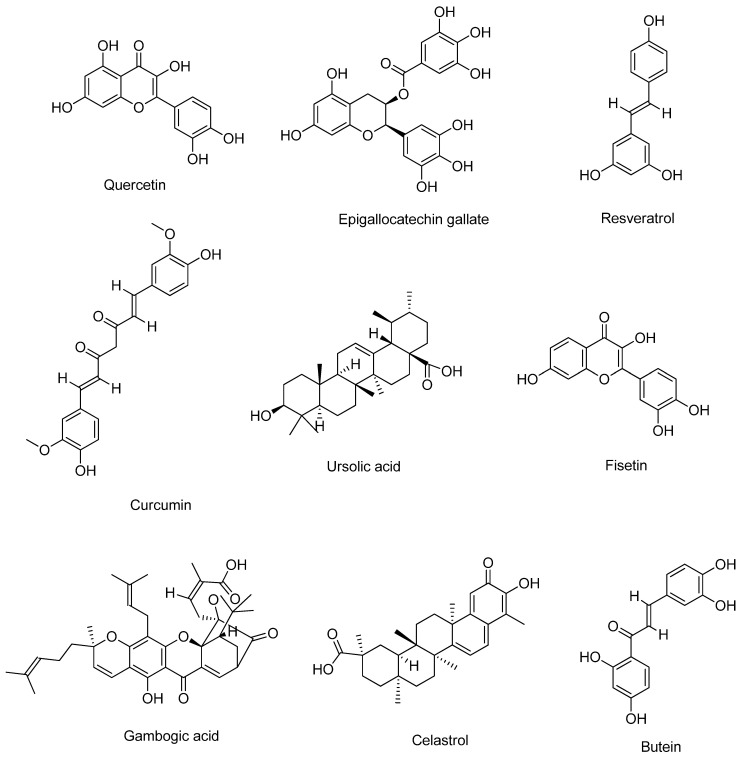
Structures of selected bioactive compounds used in the treatment or management of haematological cancers.

The mechanisms of action of selected bioactive compounds against various haematological cancers or haematological malignancies are presented in Table 1. The chemical structures of the selected bioactive compounds were searched in the PubChem and NIST Chemistry databases and redrawn with ChemDraw software (version 12.0.2) Figure 2.

## 2. Bioactive Compounds

### 2.1. Quercetin

**Figure 3:**
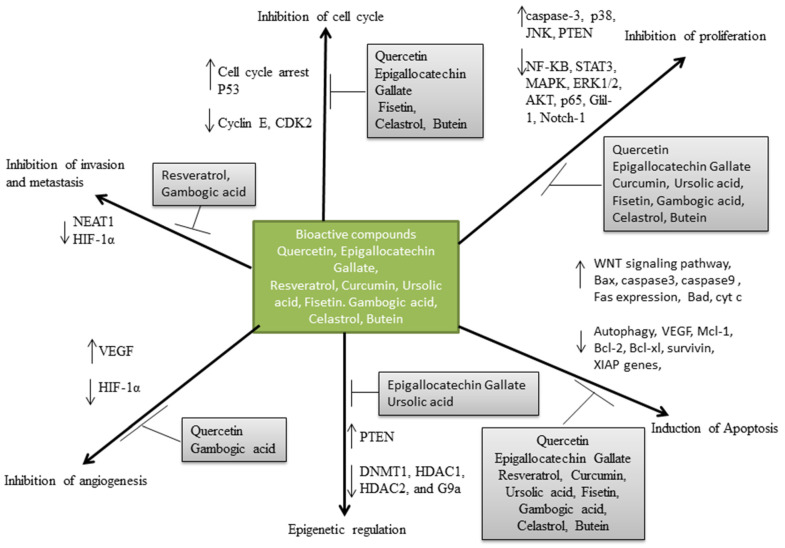
Proposed molecular mechanism of selected bioactive compounds against various types of haematological cancer models 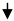 indicate downregulation and 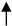 indicate upregulation.

Quercetin (3,5,7-trihydroxy-2-[3,4-dihydroxyphenyl]-4Hchromen-4-one) as shown in Figure 2, is mostly referred to as a dietary flavonoid and can be found in large amounts in vegetables such as onions, tomatoes, lettuce, capers [35,36]. This compound has been explored for its anti-oxidant, anti-bacterial, anti-inflammatory, anti-carcinogenic and anti-viral properties [36,37,38,39]. Numerous research works have sought to investigate the potential of quercetin in the treatment of haematological malignancies and their main pharmacological properties are as shown in Figure 3. Kawahara et al. [40] studied the effect of quercetin on the growth of leukaemia and lymphoma using five leukaemia cell lines (NB4, HL60, K562, Jurkat and Daudi). The WTS-1 assay revealed quercetin’s ability to inhibit the growth of the cells in a dose-dependent manner. Using a P39 cell line model, Maso et al. [32] through an MTT assay also confirmed a significant reduction in the number of viable cells in quercetin-treated cells at a concentration of 50–100 μmol/L. Additionally, after 72 h of treatment with quercetin, analysis of human 232B4 chronic lymphocytic leukaemia using MTT assay showed a decrease in proliferative capacity with an IC_50_ value of 24 µM [41]. Similar outcomes were also observed when acute myeloid leukaemia cells, MV4-11 and HL-60, were treated with quercetin [34].

This ability of quercetin to reduce the number of viable cells has been attributed to apoptotic induction, and several confirmatory studies have been carried out to establish this. A decrease in Bcl-2, Bcl-xl and Mcl-1, known as anti-apoptotic proteins, has been reported in Quercetin-treated cells [32,42,43,44]. Additionally, upregulation of pro-apoptotic proteins such as Bax, caspase-3 and caspase-9 have been discovered [32,45]. In the mitochondria of cells, the effective release of Cytochrome c and the low mitochondrial membrane potential observed in quercetin-treated cells further establish this bioactive compound as an apoptosis inductor [32,34,41,46].

In synergistic studies, quercetin and curcumin have been found to induce apoptosis in chronic myeloid leukaemia when used to treat K562 cells [26]. Likewise, quercetin has been used alongside biological cytokines such as TNF-related apoptosis-induced ligand (TRAIL) to sensitise apoptosis induction in KG-1 cells of human myeloid leukaemia [33]. Co-administration of quercetin with the drug Adriamycin resulted in the use of a lower concentration of the drug to achieve a similar inhibitory against the proliferation of primary leukemic cells [42]. Therefore, the administration of quercetin could improve the functionality and safety of already established treatment methods.

To understand the mechanism behind apoptosis induction by quercetin treatment, Alvarez et al. [47] investigated the human HL60 and U937 cell lines. They discovered the potential of quercetin to demethylate BCL2L11 and DAPK1 genes. The methylation of DAPK1 has been recognised as a contributing factor to the development of B-lymphoma, acute myeloid leukaemia and myelodysplastic syndrome [48,49]. Additionally, hypermethylation of DNA at the BCL2L11 promoter region leads to downregulation of the gene, and this contributes to the survival of chronic myeloid leukaemia [50]. Therefore, the demethylation of these pro-apoptotic genes by quercetin is central to its apoptotic induction potential. In another study, Shi et al. [34] discovered that the underlying mechanism behind the action of quercetin on MV4-11 cells was associated with the suppression of VEGF signalling, which is an important prognostic factor in the progression of acute myeloid leukaemia [51]. Inhibition of VEGF through the Akt signalling pathway was observed in quercetin-treated MV4-11 cells in a dose-dependent manner.

Another relevant phenomenon associated with the potential of quercetin in the treatment of haematological malignancies includes its autophagocytic potential. The conversion of LC3-I cells to LC3-II cells and the presence of autophagy markers such as P13K, Beclin-1, Atg5-Atg12 and Atg7 have been observed [32]. The ability of quercetin to induce cell cycle arrest [45], ERK signalling activation [52], Akt-mTOR phosphorylation [32] and the downregulation of Mcl-1, survivin and XIAP genes contribute to its potential in treating haematological malignancies [53].

In in vivo experiments, the treatment of a mouse model with 120 mg/kg of quercetin resulted in 30% inhibition of tumour growth using P39 cell lines, which was observed to be associated with activating p-ERK and p-JNK signalling pathway [32]. In another experiment, administration of the same concentration of quercetin alone to xenografted SCID mice resulted in 44% inhibition of tumour growth, while in synergy with extracts of green tea, 60% tumour inhibition was recorded [42]. In a pilot study to examine the anti-tumour effects of quercetin on patients with chronic lymphocytic leukaemia and small lymphocytic lymphoma, oral administration of quercetin resulted in varying effects in the patients under observation. However, quercetin was non-toxic and stimulated the reduction in specific disease parameters such as lymphocyte number and a decline in lactate dehydrogenase [54] (Table 1).

### 2.2. Epigallocatechin Gallate

Epigallocatechin Gallate (EGCG), a flavone-3-ol polyphenolic compound, as shown in Figure 2, can be found in green tea extracts and possesses eight different hydroxyl groups which contribute to its biological versatility [55]. It has been recognised for its radical scavenging capacity, ability to regulate gene expression, inhibit oxidative stress and anti-inflammatory properties [55,56,57]. Although green tea extract is composed of other compounds, EGCG is the most abundant and is believed to be the most effective bioactive component [58]. EGCG has been used to study different cancers, including haematological disorders, with their mechanism highlighted in Figure 3. Treatment of HL-60 and Jurkat cells with 50–100 µM of EGCG reduced the proliferation of these cells through modulation of pro-apoptotic genes and induction of differentiation [59,60,61]. Additionally, the potential of EGCG to inhibit the proliferation of retinoic-resistant acute promyelocytic leukaemia cells through mitochondria damage, ROS activation and caspase activation has been reported [62]. In addition, treatment with EGCG has led to the downregulation of epigenetic modifiers such as DNMT1, HDAC1, HDAC2 and G9a in acute myelocytic leukaemia cells [61].

Using a mouse model with acute promyelocytic leukaemia, the in vivo potential of whole extracts of green tea has been analysed. Treatment of the model with 250 mg/kg of green tea extracts for 4 days resulted in a significant reduction in the percentage of white blood cells and decreased monocyte and neutrophil levels [63]. Additionally, the number of promyelocytes, usually known to be high in leukemic cells [64], was reduced upon treatment. Similar outcomes have also been reported by Della et al. [65] using the same cell line. Furthermore, an analysis of the bone marrow and spleen of EGCG-treated cells showed an increase in the number of apoptotic cells alongside an increase in caspase-3, 8 and 9 enzyme activity [63]. Equally, an increase in caspase-3 level and Fas expression have also been recognised in T-lymphoblastic leukaemia cells [59], which is an indication of apoptotic induction [66].

However, the findings of Xiao et al. [67] deviates from this, as treatment of cells with EGCG did not activate caspase-3 and 9 in the mitochondria of chronic myeloid leukaemia cells. Although apoptosis was still induced in these cells upon EGCG treatment, this was linked to an increase in Apoptosis Inducing Factor (AIF), a phenomenon known as caspase-independent cell death [68]. Additionally, an increase in the autophagy gene, Atg5, has been observed in EGCG-treated acute promyelocytic leukaemia cells [63,69].

The anti-tumour effects of green tea extracts were also evident in the ability of the administered extracts to reduce the percentage of CD^34+^ haematopoietic progenitor cells in acute promyelocytic leukaemia cells [63]. Additionally, while the growth of bone marrow cells of chronic myeloid leukaemia patients was inhibited upon treatment with EGCG, the bone marrow of healthy donors showed resistance [67]. This variability in response, therefore, asserts the specificity of EGCG to leukemic cells. In synergy with other compounds, ECGC showed no enhanced inhibitory effect when combined with the drug imatinib but could effectively inhibit the growth of imatinib-resistant cells [67]. With all-trans retinoic acid (ARTA), EGCG increased the expression of CD15 cells, which is a neutrophil differentiator and may induce apoptosis [65].

When combined with ponatinib, the synergistic effect of both compounds altered the expression of genes associated with the G1 to S phase transition of cells, thereby altering the cell cycle [70]. In its oxidative forms, the anti-cancer properties of EGCG have also been reported in T-cell acute lymphoblastic leukaemia cell line [71]. As a therapy to support other treatments, the potential of EGCG in the prevention of graft versus host cell disease has been studied [72]. Due to the susceptibility of patients undergoing allogeneic hematopoietic stem cell transplantation to inflammatory condition, amongst others, the immunomodulatory and anti-angiogenic potential of EGCG could lead to more effective treatments (Table 1).

### 2.3. Resveratrol

The compound resveratrol (3,4,5 trihydroxystilbene) (Figure 2) is a natural polyphenol which is found in different plant species, but present in large amounts in berries, nuts, tea, skin of red grapes and pomegranates [73,74]. So far, different therapeutic benefits of resveratrol have been identified such as its neuroprotective, anti-oxidant, immunomodulatory, anti-cancer, anti-inflammatory and anti-platelet activities [75,76,77]. Widely known to be produced in plants in response to external attacks, the efficacy of resveratrol has been explored in several cancer related studies due to its ability to inhibit cell proliferation [78]. More specifically, several studies have been carried out to examine the potential of this bioactive compound as a prospective solution to certain haematological malignancies. Figure 3 highlights some of these mechanisms as reported in different research. At a concentration of 200 µmol/L, resveratrol was found to inhibit the proliferation of acute lymphoblastic leukaemia cells in a time-dependent manner, of which optimum results were obtained at 24 h [79,80,81]. In another study, 100 µM of resveratrol inhibited the proliferation of four human multiple myeloma cell lines within a period of 48 h. It produced a long-term inhibitory effect when cell colonies were observed after a 7-day incubation period [82]. The interaction between resveratrol and a long coding RNA, NEAT1, whose aberrant expression has been related to several human cancers, has also been examined in multiple myeloma cells [30]. Preceding treatment with resveratrol, the expression of NEAT1 in multiple myeloma cells was significantly high.

However, the RT-qPCR analysis revealed significant repression of this long coding RNA in the cells followed by downregulation of c-Myc and MMP-7 genes in the Wnt/β-catenin signalling pathway. The repression of NEAT1 indicates loss of proliferative and metastasis capacity of the cells [83]. miRNAs such as miR-196b and miR-1290 have also been identified as targets in the anti-tumour activity of resveratrol [84]. Comparing the action of this bioactive compound with other anti-cancer drugs such as Triptolide, Hesperidin and Polydatin, the capacity of resveratrol to inhibit the expression of NEAT1 was more pronounced than the other treatments at the same concentration [30]. Additionally, significant inhibition of G-C resistant cells through induction of caspase-3, upregulation of pro-apoptotic genes such as Bim, Bad, Bax and downregulation of Bcl-2 and Mcl-1 has been recognised [79,82]. The generation of reactive oxygen species has also been stated to be a possible mechanism through which resveratrol induces its apoptotic effect. This has been shown using diffuse large B-cell lymphoma cell lines [85]. Additionally, the repression of unfolded protein response in multiple myeloma and human Burkitt’s lymphoma has been identified using resveratrol in the treatment of haematological malignancies [30,86].

A combinatorial therapy of resveratrol and carfilzomib on multiple myeloma cell lines produced a synergistic effect, resulting in the administration of low doses of carfilzomib in inducing the same level of apoptosis in the cells [80]. Despite carfilzomib being an effective target for multiple myeloma, issues bordering drug toxicity and resistance have been identified [87]. Therefore, a combinatorial therapy such as this could make cancer cells more sensitive to the anti-tumour drug, resulting in its requirement at low concentrations that would not pose toxicity to the cells. In similar research, the overexpression of P-glycoprotein, which reduces the cytotoxicity of anti-tumour drugs, was addressed using resveratrol [88]. According to their results, treatment of chronic myeloid leukaemia cells with resveratrol and Bestatin led to the downregulation of P-glycoprotein, thereby increasing intracellular drug concentration necessary in combating multi-drug resistant malignancies. The studies of Kweon et al. [89] also produced a similar result when resveratrol was tested against doxorubicin-resistant acute myeloid leukaemia cell lines through repression of MRp-1. However, when combined with an anti-neoplastic compound such as arsenic trioxide, resveratrol did not further enhance autophagy in matured leukemic cells but enhanced the suppressive effects of arsenic trioxide on leukaemia progenitor cells [90] Table 1.

### 2.4. Curcumin 

Curcumin, (1,7-bis[4-hydroxy-3-methoxyphenyl]-1,6-heptadiene-3,5-dione), as shown in Figure 2, is a yellow-coloured compound isolated from *Curcuma longa*, commonly called turmeric [91]. Traditionally, it has been used as a medicinal herb and has been found to possess anti-oxidative, anti-inflammatory, anti-microbial and anti-cancer properties [92,93,94]. The potential of curcumin in ameliorating haematological malignancies have also been observed through different mechanisms, as summarised in Figure 3. In several in vitro experimental studies, curcumin’s effectiveness in inhibiting cell proliferation has been observed using cell lines from patients with haematological malignancies. Liao et al. [95] reported a significant inhibition in the growth of A375 human melanoma cells when the cells were treated with 80 µM of curcumin for 48 h. Lower doses of curcumin at 8.29, 18.29 and 14.25 µM have also been found to inhibit the growth of A375, MV3 and MV14 cells [96]. Additionally, a derivative of curcumin known as demethoxycurcumin has been identified for its ability to induce apoptotic induction in human myeloid leukaemia cells with IC50 values in the range of 10.3–20.4 µM [31]. In vivo, the potency of curcumin has been examined using mouse xenografts infected with acute monocytic leukaemia. A significant reduction in tumour weight was observed after administration of curcumin both at 15 mg/kg/day and 30 mg/kg/day with no adverse effect on the liver and spleen of the models used [95].

At lower doses, some studies have reported the potential of curcumin to enhance the sensitivity of certain drugs, such as doxorubicin and cytarabine, which are currently approved for treating leukaemia [97,98]. This improved sensitivity could lead to better combination therapy with minimal cytotoxic effect on the host cell. Similarly, the efficiency of chemotherapeutic agents such as methotrexate, prednisone and l-asparaginase have also been found to improve when treatment is combined with curcumin against acute lymphoblastic leukaemia cells [99]. When combined with another polyphenol, curcumin and carsonic acid synergistically induced apoptosis in acute myeloid leukaemia cell lines through selective Ca^2+^ homoeostasis in the cells [100]. Additionally, the anti-leukemic activity of etoposide in Brown Norway acute myeloid leukaemia rats has been enhanced by curcumin through intensifying production of free radicals [101].

A randomised trial with patients who were intolerant to dexamethasone showed that replacing dexamethasone with curcumin in the treatment administered decreased paraprotein load and plasmacytosis by 38% and 59%, respectively [102]. As a result, patients who are intolerant to steroid therapy can be spared from the side effects and complications that accompany the use of dexamethasone. Assessing the effect of curcumin on patients with monoclonal gammopathy of undetermined significance and those with smouldering multiple myeloma, a decrease in paraprotein load and percentage of plasma cells was observed, thereby suggesting it as a suitable treatment for delaying disease progression [103,104,105].

A qualitative assessment of the anti-proliferative capacity of curcumin revealed a significant reduction in the number of cells upon treatment with curcumin due to cell cycle arrest at the G1 phase, a decrease in the expression of cyclin D and the disruption of the mitochondria membrane potential [95]. Additionally, upregulation of caspase-3 and apoptosis-inducing factors followed by a decrease in the level of Bcl2 have been recognised as mechanisms through which apoptosis is induced in cells treated with curcumin [97]. The upregulation of Hypoxia-induced factor 1, a transcriptional factor known to be activated in cells in response to systemic oxygen levels, and downregulation of NF-kB and PARP-1 cleavage have also been observed [95,97,99].

In in vitro and in vivo studies using acute monocytic leukaemia cell lines, the effect of curcumin on MAPK and NF-kb signalling through the upregulation of p38 and JNK and downregulation of ERK2 and p-p65 has been identified, coupled with its ability to downregulate genes such as MDR1, LRP, BCRP [98,106,107,108,109]. However, Papież et al. [101] reported no correlation between curcumin and NF-kB signalling. Downregulation of specific self-renewal pathways such as Glil-1 and Notch-1 has also been identified in cell lines of Burkitt lymphoma after treatment with curcumin [110] Table 1.

### 2.5. Ursolic Acid

Ursolic acid (3 β-hydroxy-urs-12-en-28-oic acid) (Figure 2) is a cyclic triterpenoid composed of five rings and a chemical structure consisting of 30 carbon molecules [111]. It is a product found in large amounts in fruits such as cranberries, in vegetables and medicinal herbs and has been explored for its different therapeutic properties [112,113,114]. In chemopreventive studies, ursolic acid functions by suppressing cell proliferation and induction of apoptosis and this has also been observed in haematological studies, as shown in Figure 3. The treatment of four different leukaemia cell lines (HL-60, U937, Jurkat and THP-1) with a concentration of ursolic acid ranging from 6.25–25 µM showed significant suppression of cell proliferation in all the cell lines [115]. Kaewthawee and Brimson [116] investigated the effect of ursolic acid on pro-inflammatory cytokines such as IL-2 and TNF-α using Jurkat leukemic T-cells. The cell viability assay showed that a concentration of ursolic acid from 30 µM could significantly inhibit the growth of cells with an IC50 value of 32.5 µM. Additionally, ursolic acid was shown to have the capacity to suppress phytohemagglutinin-induced IL-2 and TNF-α in a concentration- and time-dependent manner. Exposure of K562 leukaemia cells to increasing concentrations of ursolic acid also resulted in a decline in the expression of MCl-1 and p-Bad proteins, resulting in a total decline in the mRNA concentration level of the cells [117]. An assessment of the capacity of ursolic acid to induce differentiation showed that in human leukaemia HL-60 cell lines, ursolic acid could induce monocytic differentiation [118]. This differentiation capacity was observed to be stimulated by activation of the ERK signalling pathway, which resulted in the upregulation of certain binding proteins in the cell. So far, in studies relating to leukaemic cells, induction of apoptosis was observed in the cells after treatment with ursolic acid, and this has been attributed to inactivation of PKB, regulation of the Akt pathway, decrease in Stat 5 a/b expression and induction of Gfi-1 protein [117,119,120,121].

Treatment with ursolic acid showed a significant downregulation of intracellular β-catenin levels in multiple myeloma RPMI-8226 cells. Further analysis also revealed that different concentrations of ursolic acid resulted in a dose-dependent reduction in the expression of target β-catenin-dependent genes such as c-Myc and cyclin D1 [122]. The anti-tumour effects of ursolic acid were examined using adult T-cell leukaemia cells [123]. Findings from the experiment revealed the anti-proliferative capacity of ursolic acid, as it was able to inhibit the proliferation of T-cell leukaemia cells in a dose-dependent manner. Analysis of cell activities showed the activation of caspase-3, 7 and 9 and a decrease in the mitochondrial membrane potential, which are important factors in initiating intrinsic apoptosis [124]. Ursolic acid has also been found to induce cell death in the peripheral blood mononuclear cells of patients with adult T-cell leukaemia and upregulate the expression of PTEN-a gene whose role in tumour suppression has been well identified [121,123]. The activity of ursolic acid on multi-drug resistant acute and chronic myelocytic leukaemia has also shown a significant inhibitory effect [125]. Therefore, this encourages the use of drugs rich in ursolic acid to combat the issue of drug resistance in haematological malignancy treatments. The anti-leukemic activity of ursolic acid has also been examined in vivo using mice inoculated with U937 cells. Xenograft sectioning and staining revealed a significant reduction in cancer cells in mice treated with ursolic acid accompanied by various signs of necrosis [119] Table 1.

### 2.6. Fisetin

Fisetin (3,3′,4′,7-tetrahydroxyflavone) (Figure 2) is a hydrophobic polyphenolic compound found in strawberry, cucumber, grape, onion, blueberry [126]. Several reports of its pharmacological properties have been reported in the literature. They include fisetin as an anti-microbial agent, anti-diabetic, anti-carcinogenic, anti-inflammatory and anti-oxidant [126,127,128]. As a natural anti-oxidant, the chemotherapeutic effect of fisetin has been examined in different cancer-related studies, including the prevention and treatment of haematological malignancies. The mechanism through which this occurs in different cells and under different conditions is as shown in Figure 3. In human K562 chronic myeloid leukaemia cells, treatment of cells with fisetin inhibited cell growth in a dose- and time-dependent manner, recording an IC50 value of 120 µM [129]. Upon further analysis of the effect of fisetin on the cells, apoptotic induction was observed to be directly proportional to the concentration of fisetin. Mitochondrial membrane depolarisation was also observed in cells treated with varying concentrations of fisetin (50, 100, 200 µM). In HL-60 acute promyelocytic cells, the inhibitory concentration of fisetin was 82 µM for a period of 48 h [129]. However, comparing this inhibition potential with another flavonoid known as hesperetin showed a better inhibitory concentration following a more extended incubation period. Using WEHI-3 mouse leukaemia cells, the IC50 value for fisetin was estimated to be approximately 24.8 µM [130], and in U266 multiple myeloma cells, 50 µM fisetin produced a significant apoptotic induction [131].

Gene expression analysis and profiling of fisetin-treated K562 and HL-60 cells revealed the regulation of genes involved in apoptosis and cell proliferation [129,132]. Specific tumour suppressor genes such as NFKBIA, PMAIP1, CDKN1A, GADD45B, TFNIP and THP1, known for inhibiting cell survival, suppressing cell growth and inducing growth arrest, were upregulated [133,134]. On the other hand, downregulation of oncogenic transcription factors implicated in leukaemia was also identified. The mechanism of fisetin-induced apoptosis has been attributed to an increase in caspase-3 activity and cell cycle arrest at S and G2 phases in K562 cells [129], G0/G1 phase arrest, increase in caspase-3, 8 and 9 activities, chromatin condensation and DNA fragmentation in WEHI-3 cells [130]. Additionally, the downregulation of Bcl-2 and Mcl-1, which are anti-apoptotic proteins and upregulation of pro-apoptotic proteins such as Bax and Bad have been recognised [131].

However, the research carried out by Klimaszewska-Wiśniewska et al. [135] revealed that careful consideration must be observed in using fisetin in in vivo experimental studies. This is because, even at low, clinically recommended concentrations, the activity of fisetin in some cells, such as chronic myeloid leukaemia, might be antagonistic. The experimental outcomes of the research showed a negligible apoptotic induction in cells treated with a low concentration of fisetin, followed by modulation of markers associated with metastasis in cells Table 1.

### 2.7. Gambogic Acid

Gambogic acid also known as Beta Guttiferin, having the structure shown in Figure 2, is a natural resin secreted from a tree known as *Garcinia hanburyi*. It is the most common family of a group of compounds known as xanthone and is widely known for its pharmacological properties [136]. In seeking natural treatments for haematological malignancies, gambogic acid’s potential has also been explored and different pharmacological properties have been discovered, as shown in Figure 3. The treatment of human myeloma cell lines with a combination of Bortezomib [BTZ] and gambogic acid resulted in a synergistic anti-proliferative capacity where the inhibition rate was recorded to be approximately 73% [137]. This result reflects that co-administration of BTZ and gambogic acid enhances the drug’s apoptosis-inducing effect, thereby improving its inhibitory potential. Additionally, this synergy indicates the potential of adopting low concentrations of BTZ in treating multiple myeloma to eradicate challenges associated with drug resistance and dose toxicity. Imatinib-resistant chronic myeloid leukaemia cell lines also responded to treatment with gambogic acid as cell viability was effectively inhibited within a 48 h incubation period with concentrations ranging between 0.32, 0.35 and 0.40 µM [138]. In the same study, mononuclear cancer cells from imatinib-sensitive and imatinib-resistant patients, including xenografted tumours, showed sensitivity upon treatment with gambogic acid. In large B-cell lymphoma cells, gambogic acid significantly inhibited cell proliferation at minimal concentrations of 0.16 to 0.30 µM [139]. When gambogic acid was examined against K562 human leukaemia cell lines, a 63% inhibitory potential was recorded [140].

The mechanism of NF-kB downregulation and caspase activation by gambogic acid has been attributed to proteasome inhibition in B-cell lymphoma cells and chronic myeloid leukaemia cells [138,139]. Additionally, a significant reduction was observed in AKT, Erk1/2 and Stat 5 phosphorylation showing the effect of gambogic acid on major signalling pathways involved in cell growth. Analysis of proteins in the cells treated with gambogic acid showed the regulation of pro-apoptotic and anti-apoptotic proteins such as PARP, p53, Bcl-2, Bax, caspase-3, cell cycle arrest at the G2/M phase [137,140,141]. The key transcription factor (HIF-1α) known to be activated in individuals diagnosed with multiple myeloma was also suppressed after in vitro treatment of cells with gambogic acid [142,143]. This was associated with a regulation in miR-21, whose aberrant expression has been implicated in hypoxic conditions relating to multiple myeloma [144]. In human acute T-cell leukaemia cells, gambogic acid induced autophagy through downregulation of the β-catenin signalling pathway [145].

Additionally, through the suppression of the CXCR4 signalling pathway, Pandey et al. [146] discovered the effect of gambogic acid in addressing osteoclastogenesis. The inhibition of the development of this condition is crucial in overcoming one of the hallmarks of multiple myeloma [147]. The treatment of acute myeloid leukaemia cells with varying concentrations of gambogic acid has also shown the induction of differentiation in the cells [148]. In the study of Yang et al. [149], gambogic acid was observed to downregulate SIRT1 in multiple myeloma cells by accumulating reactive oxygen species (Table 1).

### 2.8. Celastrol

Celastrol is a pentacyclic tetrapenoid (Figure 2) extracted from a traditional Chinese medicine herb known as *Tripterygium wilfordii.* Over the years, celastrol has been used in the treatment of different conditions such as cancer, obesity and neurodegenerative diseases [150,151,152]. Due to its effectiveness as a proteasome inhibitor, it is used in treating multiple myeloma [153,154]. In treating haematological malignancies with celastrol, different mechanisms have also been observed, as shown in Figure 3. Using three different human myeloma cell lines and the tumour tissue of a mouse xenograft model, celastrol elicited anti-proliferative activity in the cells in a dose-dependent manner [155]. This was evidenced by an increase in apoptotic cells and cell cycle arrest observed at the G0/G1 phase. Additionally, the caspase, trypsin and chymotrypsin-like proteasome activities were effectively inhibited in the cells and the in vivo studies carried out in the tumour tissue significantly reflected the impact of celastrol on proteasome activity. The inhibitory potential of celastrol has also been demonstrated by its ability to downregulate Bcr-Abl genes, which have been identified as additional oncogenes in chronic myeloid cells, thereby serving as a reliable therapeutic target [156]. These genes modulate a cascade of events whose eventual target is to facilitate the escape of the cells from apoptosis [157,158]. Additionally, apoptosis induction through the regulation of apoptotic proteins observed in chronic myelogenous cells resistant and sensitive to treatment with imatinib have also been identified in celastrol-treated cells.

In other studies, celastrol has been observed to effectively suppress the proliferation of multiple myeloma cells through downregulation of the NF-kB and STAT3 pathway [151,159,160,161]. The impact of celastrol on these pathways resulted in events such as the upregulation of caspase-3 and regulation of pro-apoptotic and anti-apoptotic genes, Bcl-2, Bcl-Xl, survivin, XIAP and Mcl-1. The chemotherapeutic potential of drugs such as thalidomide and bortezomib in treating multiple myeloma cells has also been shown to improve in the presence of celastrol, thereby countering issues associated with drug resistance and improving chemosensitivity [151,160]. In combination with another naturally derived product known as epigallocatechin gallate, celastrol exhibited potent biological activities essential to treating leukaemia [162]. Metabolomic studies have also identified that the pro-apoptotic properties of celastrol in human acute promyelocytic leukaemia cells may be due to the activation of the p-53 mitochondrial pathway caused by notable changes in uridine levels in the cells [163] Table 1.

### 2.9. Butein

Butein (3,4,2′,4′-tetrahydroxychalcone) (Figure 2) is a flavonoid isolated from plants such as *Toxicodendron vernicifluum* and has found application as an anti-oxidant, antibacterial, anti-inflammatory and anti-cancer agent [164,165]. In haematological malignancies, the potential of butein has also been examined and different pharmacological properties have been observed, as shown in Figure 3. In acute lymphoblastic leukaemia cells, the effect of butein has been observed on the proliferation of cells and the inhibition of cell cycle progression due to a downregulation of cyclin E, CDK2 and the upregulation of caspase-3 expression [166]. These changes in the cells were reported to be due to the capacity of butein to regulate the FOXO3a signalling pathway, a pathway known for inducing apoptosis in cells [167,168]. Additionally, the higher levels of telomerase activity exhibited in leukaemia cells were reported to be significantly downregulated upon treatment of cells with butein [169]. Through Western blot and qPCR analysis, the transcriptional level of hTERT gene was significantly lower, which was later observed to be associated with a downregulation of c-Myc gene transcription and Akt-dependent phosphorylation. The regulation of other signalling pathways such as NF-kB, AP-1, P53 and Akt has also been recognised as mechanisms through which butein exhibits its pro-apoptotic potential [170,171]. Confirmatory in vivo studies have shown the reduction in tumour growth in leukemic mice treated with butein [170]. The synergistic potential of butein has also been explored in combination with Tumour Necrosis factor Apoptosis Related Ligand (TRAIL) [172]. The Trail-resistant leukaemia cells were sensitised to undergo apoptosis due to the presence of butein in the treatment, evidenced by an increase in caspase-3 activity Table 1.

**Table 1:** Different bioactive compounds used in the treatment of haematological malignancies, their mechanism of action, experimental models and conditions of usage.

Bioactive Compound	Experimental Models	Condition	Effective Dose	Mechanism	References
Quercetin	NB4, HL60, K562, Jurkat and Daudi	Leukaemia and lymphoma	50 µmol/L	Apoptosis through activation of the Wnt signalling pathway	[40]
Quercetin	P39	Leukaemia	50, 100 µmol/L	Apoptosis through an upregulation of pro-apoptotic proteins and downregulation of anti-apoptotic proteins, autophagy, Akt-mTOR phosphorylation, tumour inhibition	[32]
Quercetin	Human232b4	Chronic lymphocytic leukaemia	24 µM	Decrease in the proliferative capacity of cells due to activation of caspase-3 and cell cycle arrest	[41]
Quercetin	MV4-11, HL-60	Acute myeloid leukaemia	Dose-dependent	Apoptosis via downregulation of vascular endothelial growth factor signalling	[34]
Quercetin	HL-60, U937	Leukaemia	50 µmol/L	Demethylation of pro-apoptotic genes, BCL2L11 and DAPK1	[47]
Quercetin	U937	Leukaemia	120 µM	Down regulation of Mcl-1, survivin and XIAP genes	[44]
Quercetin	Patients	Chronic lymphocytic leukaemia	500 mg twice daily for 3 months	Reduction in lymphocyte number and a decline in lactate dehydrogenase	[54]
Quercetin and Curcumin	K562	Chronic myeloid leukaemia	Dose-dependent	Apoptosis	[26]
Quercetin and TRAIL	KG-1	Human myeloid leukaemia	105.6 µM	Sensitise TRAIL to induce apoptosis	[33]
Quercetin and Adriamycin	HL-60 xenografts	Human leukaemia	100 µM	Lower concentration of Adriamycin to inhibit proliferation of cells	[42]
Quercetin and Green Tea	HL-60 xenografts	Human leukaemia	120:100 mg/kg	Tumour inhibition	[42]
Epigallocatechin Gallate	Leukemic mice	Acute promyelocytic leukaemia	200 µM	Apoptosis, tumour inhibition via reduction in the number of promyelocytes and reduction in CD^34+^ haematopoietic progenitor cells	[63]
Epigallocatechin Gallate	Bcr/Abl+	Chronic myeloid leukaemia	Dose-dependent	Caspase-independent apoptosis through upregulation of apoptosis-inducing factor	[67]
Epigallocatechin Gallate	NB4, NB4-R1, NB4-R2	Promyelocytic leukaemia	200 µM	Mitochondria damage, ROS activation, caspase activation	[62]
Epigallocatechin Gallate	Jurkat cells	T lymphoblastic leukaemia	250 mg/kg	Apoptotic induction through increase in caspase-3 level and Fas expression	[59]
Epigallocatechin Gallate	NALM-6	Acute myelocytic leukaemia	45 µM	Downregulation of epigenetic modifiers such as DNMT1, HDAC1, HDAC2, G9a	[61]
Epigallocatechin Gallate + ponatinib	K562	Chronic myeloid leukaemia	87.13 nM and 50 µM	Cell cycle arrest	[70]
Resveratrol	Human multiple myeloma tissue-U266 and LP-1	Multiple myeloma	Dose-dependent	Repression of NEAT1 and down regulation of c-Myc and MMP-7 genes	[30]
Resveratrol	Molt-4 and Jurkat	T-cell acute lymphoblastic leukaemia	75 µM	Apoptosis and autophagy through regulation of pro-apoptotic and anti-apoptotic genes	[79]
Resveratrol + Carfilzomib	LP-1, U266	Multiple myeloma	200 µM	Modulation of metabolism, stress and apoptosis through ROS generation	[80]
Resveratrol + Bestatin	K562	Chronic myeloid leukaemia	10 µM	Downregulation of P-glycoprotein	[88]
Curcumin	A375	Acute monocytic leukaemia	80 µM	Reduction in tumour weight, disruption of mitochondria membrane potential	[95]
Curcumin + doxorubicin	REH and RSV	Acute lymphoblastic leukaemia	100 µM	Enhanced sensitivity of drug to apoptosis induction	[97]
Curcumin+ cytarabine	Bone marrow samples	Acute myeloid leukaemia	12.41;3.1 µM	Synergistic effect which enhanced the anti-proliferative capacity of cytarabine, Downregulation of MDR genes	[98]
Curcumin	Multiple myeloma patients	Multiple myeloma	3–4 g daily for 3 months	Decrease in paraprotein load and plasmacytosis	[102]
Curcumin	SH-1	Human monocytic leukaemia	32.40 µM	Alteration of MAPK and MMP signalling	[109]
Curcumin	BL41-3, DG-75, THP1	Burkitt Lymphoma	Dose-dependent	Downregulation of Glil-1, Notch 1	[110]
Ursolic acid	HL-60, U937, Jurkat and THP-1	Leukaemia	25 µM	Suppression of cell proliferation	[115]
Ursolic acid	Jurkat	Leukaemia	30 µM	Suppression of phytohemagglutinin induced IL-2 and TNF-α	[116]
Ursolic acid	K562	Leukaemia	Dose-dependent	Downregulation of MCl-1 and p-Bad proteins	[117]
Ursolic acid	HL-60	Leukaemia	60 µmol/L	Monocytic differentiation through activation of the ERK signalling pathway	[118]
Ursolic acid	RPMI-8226	Multiple myeloma	40 µM	Downregulation of intracellular β-catenin levels and reduction in the expression of target β-catenin dependent genes such as c-Myc and cyclin D1	[122]
Fisetin	K562	Myeloid leukaemia	163 µM	Apoptosis and mitochondrial membrane depolarisation through increase in caspase-3 activity and cell cycle arrest at S and G2 phases	[129]
Fisetin	U266	Multiple myeloma	60 µM	Downregulation of Bcl-2, Mcl-1 and upregulation of Bax and Bad	[131]
Gambogic acid	KBM5, K562	Imatinib-resistant chronic myeloid leukaemia	0.40 µmol/L	Sensitivity of cells to treatment	[137]
Gambogic acid + Bortezomib	MM.1S	Human myeloma	0.9 µM GNA + 4.0 Nm BTZ	Enhancing the apoptosis-inducing effect of the drug through NFkB downregulation and caspase activation	[138]
Gambogic acid	DLBCL cell lines and mouse models	B-cell lymphoma	0.30 µM	Proteasome inhibition resulting in NF-kB downregulation and caspase activation	[138]
Gambogic acid	U266	Multiple myeloma	Dose-dependent	Suppression of HIF-1α	[143]
Celastrol	MM.1S, MM.1R, U266	Human myeloma	500 nM	Induction of anti-proliferative activity, cell cycle arrest and proteasome inhibition	[155]
Celastrol	KBM5	Chronic myeloid leukaemia	525.4 nM	Downregulation of Bcl-Abl genes	[156]
Celastrol	U266, RPMI8226	Multiple myeloma	Dose-dependent	Downregulation of the NF-kB and STAT3 pathway	[151]
Celastrol	HL-60	Human acute promyelocytic leukaemia	0.55 µM	Activation of the p-53 mitochondrial pathway with notable increase in uridine levels	[163]
Butein	RS4-11, MOLT-4	Acute lymphoblastic leukaemia	100 µM	Downregulation of cyclin E, CDK2 and the upregulation of caspase-3 expression through the FOXO3a signalling pathway	[166]
Butein	HTL-V1 infected T-cells	Adult T-cell leukaemia	7.0 µM	Apoptosis induction through the regulation of other signalling pathways such as NF-kB, AP-1, P53 and Akt	[170]

The anti-inflammatory, anti-oxidant and biological effect of polyphenols can be attributed to their structure. They elicit anti-oxidant effects through the presence of multiple hydroxyl groups. The hydroxyl groups neutralise free radicals, producing stable compounds and preventing free radical chain reactions that would have resulted in inflammation and damage to cells [173]. The position, degree of hydroxylation of the compound and the number of hydroxyl aromatic rings dictates the level of anti-oxidant activity it will possess. The anti-inflammatory effect of polyphenols is dependent on their anti-oxidant abilities, suppression of inflammatory signalling pathways and interfering with oxidative stress signalling [174,175].

## 3. Conclusions

In this study, the selected bioactive compounds quercetin, ursolic acid, fisetin, resveratrol, epigallocatechin gallate, curcumin, gambogic acid, butein and celastrol showed promise as potent novel therapeutic activities against haematological cancer types in in vivo and in vitro studies. These biologically active compounds have the capacity to exert their anti-cancer potentials against haematological cancers via different molecular mechanisms such as downregulation or upregulation of signalling pathways, induction of apoptosis, inhibition of cell proliferation, inhibition of angiogenesis, epigenetic regulations, inhibition of invasion and metastasis, proteosome inhibition, increasing the sensitivity of cells to treatment and blockage of cell cycles. They also showed promise in working in synergy with already established chemotherapeutic drugs to increase their efficacy. This review provides strong evidence that the studied bioactive compounds are effective in reducing the risk of haematological cancers in both the in vivo and in vitro experimental models. The study showed that quercetin is the most studied bioactive compound in the treatment of haematological cancer as it shows the greatest potentials for the treatment of leukaemia. However, further studies are required to fully elucidate various signalling pathways and detail mechanisms involved in the use of these bioactive compounds in the prevention, treatment and management of haematological cancers. Additionally, studies on increasing the bioavailability and pharmacokinetics of these bioactive compounds should be undertaken, possibly via nanoencapsulation. Finally, there is need for more human clinical studies on these bioactive compounds to establish their effective doses or concentrations and optimal plasma levels for the treatment of haematological cancers.

## Data Availability

No applicable.

## Abbreviations

Akt	AKT serine/threonine kinase 1
ALL	Acute lymphoblastic leukaemia
AMPK	Adenosine Monophosphate-Activated Protein Kinase
AP-1	Activator Protein 1
Atg	Autophagy related
Bad	BCL2 Associated Agonist of Cell Death
Bax	Bcl-2 Associated X-protein
Bcl-2	B-cell lymphoma 2
BCL2L11	Bcl-2-like protein 11
Bcl-xL	B-cell lymphoma- extra large
Bcr-Abl	Breakpoint cluster region and Abelson murine leukaemia viral oncogene homologue
BCRP	Breast cancer resistance protein
Bim	BCL2-interacting mediator of cell death
CDK2	Cyclin-dependent kinase 2
CDKN1A	Cyclin-Dependent Kinase Inhibitor 1A]
c-Myc	Cellular-Master Regulator of Cell Cycle Entry
CXCR4	C-X-C motif receptor 4
DAPK1	Death-Associated Protein Kinase 1
DNMT1	DNA methyltransferase
ERK	Extracellular signal-regulated kinase
Fas	Fas Cell Surface Death Receptor
FOXO3a	Forkhead box transcription factors
GADD45B	Growth Arrest and DNA Damage Inducible Beta
Gfi-1 protein	Growth Factor Independent 1 Transcriptional Repressor
Glil-1	Glioma-associated oncogene homologue 1
HDAC	Histone deacetylase
hTERT	human Telomerase reverse transcriptase
IL-2	Interleukin-2
JAK/STAT	Janus Kinase/Signal Transducer and Activator of Transcription
JNK	c-Jun N-terminal kinases
LC3-I	Microtubule-associated protein 1A/1B-light chain 3-I
LC3-II	Microtubule-associated protein 1A/1B-light chain 3-II
LRP	Low-density lipoprotein receptor-related protein 1
MAPK	Microtubule-Associated Protein Kinase
Mcl-1	Myeloid cell leukaemia-1
MDR1	Multidrug resistance gene
miR-1290	MicroRNA 1290
miR-196b	MicroRNA 196b
miR-21	MicroRNA 21
MMP	Mitochondrial membrane potential
MMP-7	Matrix metalloproteinase
MRp-1	Multidrug resistance protein 1
mTor	The mechanistic target of rapamycin
NEAT1	Nuclear Paraspeckle Assembly Transcript 1
NF-kB	Nuclear Factor-Kappa B
NFKBIA	NFKB Inhibitor Alpha
Notch-1	Neurogenic locus notch homolog protein 1
PARP-1	Poly-Adenosine Diphosphate Ribose Polymerase-1
p-ERK	Phospho-extracellular signal-related kinase
PI3K	Phosphoinositide 3-kinases
PI3K-AKT	Phosphatidylinositol-3-Kinase
p-JNK	Protein and c-jun NH2-terminal kinase
PKB	Protein kinase B
PMAIP1	Phorbol-12-Myristate-13-Acetate-Induced Protein 1
PTEN	Phosphatase and Tensin Homolog
ROS	Reactive oxygen species
SCID	Severe combined immunodeficiency
Stat 5 a/b	Signal transducer and activator of transcription 5
TNF-α	Tumour Necrosis factor alpha
TRAIL	TNF-related apoptosis-induced ligand
VEGF	Vascular endothelial growth factor
Wnt/β	Wnt/beta-catenin
WST	Water soluble tetrazolium salt
XIAP	X-linked inhibitor of apoptosis protein
